# Comparative evaluation of *Calotropis gigantea* latex and sodium hypochlorite as tissue-dissolving agents – an *in vitro* study on concentration and time-dependent proficiency

**DOI:** 10.2340/biid.v13.45992

**Published:** 2026-05-06

**Authors:** Megavarnan Ravi, Praveena Annadurai, Ramlakhan Gupta

**Affiliations:** a Sathyabama Institute of Science and Technology, Sathyabama Dental College and Hospital, Chennai, India; b Department of Conservative Dentistry and Endodontics Sathyabama Dental College and Hospital, Chennai, India

**Keywords:** *Calotropis gigantea*, tissue dissolution, sodium hypochlorite, endodontic irrigant, herbal extract, biocompatibility

## Abstract

**Background:**

Complete removal of organic tissue and microorganisms from the root canal system is essential for successful endodontic therapy. Sodium hypochlorite (NaOCl) remains the gold standard irrigant for its tissue-dissolving and antimicrobial properties, but its cytotoxicity and adverse effects have encouraged exploration of safer herbal alternatives. *Calotropis gigantea* (milkweed) latex contains proteolytic enzymes such as calotropin and calotoxin, which may offer effective organic tissue dissolution with improved biocompatibility.

**Aim:**

To evaluate and compare the tissue-dissolving potential of *C. gigantea* latex at two concentrations (5 and 10%) with 5.25% NaOCl at different exposure times.

**Methodology:**

An *in vitro* experimental study was conducted on pulp tissues extracted from freshly removed human teeth (*n* = 30). Samples were equally divided into three groups: 5% *C. gigantea* latex, 10% *C. gigantea* latex, and 5.25% NaOCl. Tissue samples were immersed for 10 minutes and 15 minutes, and weight reduction was measured using an analytical balance. Data were analysed using one-way analysis of variance and Tukey’s *post hoc* test with a significance level of *p* < 0.05.

**Results:**

The mean initial tissue weights were comparable among groups (*p* = 0.656). After 10 minutes, significant differences in weight reduction were observed (*p* < 0.001). Tukey’s *post hoc* test showed that 10% *C. gigantea* latex dissolved significantly more tissue than both 5% latex and NaOCl (*p* < 0.001). After 15 minutes, this difference persisted (*p* < 0.001). The 10% latex achieved the highest mean weight loss at both intervals, while no significant difference was observed between 5% latex and NaOCl.

**Conclusion:**

The 10% *C. gigantea* latex solution showed higher tissue-dissolving ability compared to 5.25% NaOCl under static *in vitro* conditions. Clinical applicability requires further evaluation of activation methods, cytotoxicity, dentin interaction, and antimicrobial efficacy.

## Introduction

Successful endodontic therapy depends on the complete elimination of organic debris, necrotic tissue and microorganisms from the root canal system. The tissue-dissolving property of the endodontic irrigants plays a vital role in achieving chemo-mechanical debridement and ensures long-term success of treatment [1]. Removal of organic tissue remnants from the canal enhances irrigant penetration into canal irregularities, thereby reducing the bacterial load and improving the adaptation of obturating materials [2].

Sodium hypochlorite (NaOCl) is hailed as the gold standard for its dual ability to dissolve organic tissues and act as a broad-spectrum antimicrobial agent [3]. But its usage is limited due to its cytotoxic property to periapical tissues, unpleasant odour, taste, corrosion of instruments and risk of complications when extruded beyond the apex. Exposure to NaOCl also reduces the dentin flexural strength and adversely affects the sealer bonding [4, 5]. These limitations have prompted the exploration of natural and herbal alternatives that are safer and more biocompatible irrigation solutions [6].

Plant derivatives of *Sapindus mukorossi* (soapnut) and *Allium sativum* (garlic) have been explored for their tissue-dissolving properties [7, 8]. The protein-denaturing property and proteolytic properties of these materials help disrupt organic matter and inhibit endodontic pathogens, respectively [9, 10]. Among these, *Calotropis gigantea* (crown flower/milkweed) has garnered attention due to its proteolytic enzymes such as calotropin, calotoxin and uscharin and phytochemicals that possess antimicrobial and protein-degrading properties. These compounds facilitate dissolution of biological tissue and demonstrate better safety and biocompatibility compared to conventional chemical irrigants due to their enzymatic and plant-derived composition [11]. A study by Sharma et al. showed the anti-cariogenic and anti-bacterial potential of *C. gigantea* extract in the oral cavity. Its natural origin and ease of extraction make it a cost-effective alternative for clinical translation [12]. Thus, the current study was carried out to evaluate the tissue-dissolving potential of *C. gigantea* latex in comparison with NaOCl under varying concentrations and exposure times. The objectives of this study were to (1) assess the tissue-dissolving ability of *C. gigantea* at different concentrations, (2) analyse the influence of time on tissue-dissolving capacity, (3) compare its dissolution efficacy with that of NaOCl and (4) determine the optimal concentration and exposure time for maximum tissue dissolution.

## Materials and methods

### Study design

This was an *in vitro* study designed to evaluate and compare the tissue-dissolving potential of *C. gigantea* latex and NaOCl under varying concentrations and exposure times. Ethical approval was obtained from the Institutional Ethics Committee (Approval No: 471/IRB-IBSEC/SIST).

### Sampling methods

A purposive sampling method was used to select suitable tooth samples for the study.

### Sample size calculation

Sample size was calculated a priori using G power software with analysis of variance (ANOVA) (fixed effect omnibus, one-way) with an effect size of 0.6, alpha error probability of 0.05 and power of 0.80. Since no prior studies have evaluated the tissue-dissolving ability of *C. gigantea* latex, the effect size estimation was based on previous *in vitro* investigations that compared herbal irrigants with NaOCl. These studies have demonstrated substantial intergroup differences in biological outcomes, including pulp tissue dissolution and microbial reduction [13, 14]. Therefore, a large effect size of 0.6 was assumed to prevent the underestimation of sample size. The total sample size was calculated as 30 samples with an actual power of 0.8004.

## Materials used

Fresh latex of *C. gigantea* was collected aseptically from mature plants and used for the preparation of the experimental irrigant solutions. Commercially available NaOCl solution (5.25%; Safe Endo, India) was used as the control irrigant.

Freshly extracted human permanent teeth (premolars and third molars) were obtained from the Department of Oral and Maxillofacial Surgery of our institution.

Tooth sectioning was performed using a double-sided diamond disc (C0_2_; Jinguang, China) under continuous sterile saline irrigation to prevent overheating and damage to the tooth and pulp tissue. Sterile saline solution was also used for rinsing and cleaning of specimens.

Phosphate-buffered saline (PBS) (SRL 10X Phosphate Buffered Saline, Generic, India) was used for the storage of the cleaned teeth to maintain physiological pH and hydration prior to experimentation.

A high-precision digital analytical balance (Wensar 600 g capacity; 0.0001 g precision; Wensar, India) was used to measure the weight of pulp tissue samples before and after immersion in the experimental solutions.

### Collection and preparation of plant latex

*C gigantea* latex contains multiple bioactive and toxic constituents, including cardiac glycosides (cardenolides such as calotropin, calotoxin and uscharin), steroidal compounds, resins and alkaloids. Among these, cardiac glycosides and steroidal derivatives constitute the primary toxic fraction responsible for cytotoxic and haemolytic effects. Detoxification was therefore aimed at selectively removing these non-polar toxic constituents while preserving the water-soluble proteolytic enzyme fraction responsible for tissue dissolution. However, no direct chemical quantification or enzymatic activity assay was performed.

Fresh latex was collected aseptically from mature plants and allowed to sun-dry until complete dehydration. Analytical grade chloroform (100% v/v; LDD Bioscience, India) [15] was selected as the extraction solvent because cardiac glycosides and steroidal toxins are predominantly non-polar and lipid-soluble, making chloroform more suitable for their selective extraction compared to polar solvents. The dried latex was immersed in chloroform at a ratio of 1g per 10 mL and maintained for 24 hours at room temperature to allow adequate extraction of toxic constituents. After extraction, the chloroform phase was discarded, and the residue was air-dried in a fume hood until complete solvent evaporation was achieved. The absence of solvent odour and visible residue served as qualitative confirmation of solvent removal. After complete evaporation, a solid detoxified latex residue remained. This residue was carefully collected by scraping it from the container using a sterile spatula and transferred into sterile vials. The collected residue was then weighed and reconstituted in ethanol to prepare 5 and 10% (w/v) latex solutions for experimental use.

### Tooth collection and preparation

Freshly extracted third molars and premolars indicated for non-therapeutic extraction were collected. The extracted teeth with intact crown and root structure were obtained from the Department of Oral and Maxillofacial Surgery of our institution. All specimens were anonymised prior to inclusion in the study. Teeth with evidence of infection, inflammation, or with any pathological conditions such as caries, cysts or root resorption and impacted third molars were excluded from the study. Immediately after extraction, the teeth were cleaned with saline to remove debris. Adherent soft tissue remnants were carefully removed using a sterile scaler and curette without damaging the tooth structure. The specimens were then immersed in sterile saline solution for further cleaning and visually inspected to confirm the absence of residual debris. Following this, the teeth were transferred to PBS at 4°C to maintain tissue integrity. PBS was selected because it maintains physiological pH and osmolarity, thereby preserving hydration and structural stability of dental tissues without introducing chemical agents that could interfere with the tissue-dissolving properties of the experimental solutions. Prior to experimentation, specimens were brought to room temperature and re-examined for suitability.

### Pulp tissue preparation

Teeth were sectioned vertically from crown to root using a diamond disc under continuous saline irrigation to prevent heat generation. Pulp tissue was gently extirpated using saline tweezers and transferred to sterile containers. Each pulp tissue sample was precisely weighed using a digital analytical balance.

### Equipment

All specimens were stored in sterile plastic containers of 10 mL capacity (Spylx, India). Tissue samples were weighed using a high-precision digital analytical balance (Wensar 600 g/0.0001 g; Wensar, India). The balance was calibrated prior to each measurement according to the manufacturer’s instructions to ensure accuracy and reproducibility. Sterile micropipettes were used for precise dispensing and removal of experimental solutions.

### Study groups

The samples (*n* = 30) were divided into three groups based on the irrigant used. Group I was *C. gigantea* latex with 5% concentration, Group II included *C. gigantea* latex with 10% concentration and Group III (positive control) was 5.25% NaOCl solution. The concentrations of 5 and 10% were selected as a part of an exploratory dose-dependent experimental design, as there is currently no established standard concentration of *C. gigantea* latex for use as an endodontic irrigant in the literature. A lower concentration (5%) was chosen to minimise potential toxicity and preserve enzymatic activity. While a higher concentration (10%) was included to assess whether increased enzyme availability would enhance tissue dissolution efficacy. Each group with 10 samples was exposed to their respective irrigant at 10-minute and 15-minute intervals to evaluate the concentration and time-dependent tissue dissolution effect.

### Statistical analysis

Data obtained from the study were analysed using IBM SPSS Statistics for Windows, Version 27.0 (IBM Corp., Armonk, NY, USA). Descriptive statistics were calculated for all variables. The normality of data distribution was assessed using the Shapiro-Wilk test as the sample size in each group was less than 50. Since the data followed a normal distribution, intergroup comparisons were performed using one-way ANOVA. *Post hoc* pairwise comparisons were carried out using Tukey’s honestly significant difference (HSD) to identify differences between individual groups. A significance level of *p* < 0.05 was considered statistically significant.

## Results

The descriptive statistics showed that the mean initial weights of all samples were comparable across all the experimental groups. The highest reduction in mean weight was observed in 10% solution of the *C. gigantea* latex group (Table 1).

**Table 1:** Descriptive statistics of pulp tissue weight (mg) at baseline and after immersion in different irrigants.

Time interval	Irrigant	Mean (mg)	Standard deviation	Standard error	95% Confidence interval (lower–upper)
Initial weight	5% *C. gigantea* latex(*n* = 10)	0.1350	0.0017	0.0006	0.1337–0.1363
	10% *C. gigantea* latex(*n* = 10)	0.1347	0.0013	0.0004	0.1338–0.1356
	5.25% Sodium hypochlorite(*n* = 10)	0.1343	0.0016	0.0005	0.1331–0.1355
After 10 minutes	5% *C. gigantea* latex(*n* = 10)	0.0981	0.0033	0.0010	0.0957–0.1005
	10% *C. gigantea* latex(*n* = 10)	0.0577	0.0038	0.0012	0.0550–0.0604
	5.25% Sodium hypochlorite(*n* = 10)	0.0993	0.0051	0.0016	0.0957–0.1029
After 15 minutes	5% *C. gigantea* latex(*n* = 10)	0.0531	0.0035	0.0011	0.0506–0.0556
	10% *C. gigantea* latex(*n* = 10)	0.0241	0.0030	0.0009	0.0220–0.0262
	5.25% Sodium hypochlorite(*n* = 10)	0.0637	0.0111	0.0035	0.0558–0.0716

One-way ANOVA analysis revealed no significant difference in the initial weights of all three groups (*p* = 0.656). After 10 minutes of immersion, a statistically significant difference in sample weight was observed (*F* (2,27) = 328.32, *p* < 0.001). Tukey’s *post hoc* analysis showed that the 10% *C. gigantea* latex solution had significantly greater weight reduction compared to 5% latex and 5.25% NaOCl (*p* < 0.001), and no significant difference was observed between 5% latex and NaOCl groups (*p* = 0.794)

After 15 minutes, a significant difference persisted (*F* (2,27) = 87.16, *p* < 0.001), with the 10% latex group showing maximum weight loss. All pairwise comparisons were significant except between 5% latex and NaOCl group (*p* = 0.006) (Tables 2 and 3).

**Table 2:** One-way ANOVA comparing the mean tissue weight among irrigants at different time intervals.

Parameter	Source	SS	df	MS	*F*	*P*
Initial weight	Between groups	0.001	2	0.0005	0.43	0.65
	Within groups	0.032	27	0.0012		
	Total	0.033	29			
After 10 minutes	Between groups	0.011	2	0.0055	328.32	< 0.001*
	Within groups	0.000	27	0.0004		
	Total	0.012	29			
After 15 minutes	Between groups	0.008	2	0.0040	87.16	< 0.001*
	Within groups	0.001	27	0.000		
	Total	0.010	29			

* *P* < 0.05 indicates statistical significance. SS: sum of squares; df: degrees of freedom; MS: mean square; *F*: *F*-statistic (ANOVA test value). ANOVA: analysis of variance.

**Table 3:** Tukey HSD post hoc comparison between irrigants.

Dependent variable	(I) Irrigant	(J) Irrigant	Mean difference (I–J)	Standard error	*P*	95% Confidence interval (lower–upper)
After 10 minutes	5% *C. gigantea* latex	10% *C. gigantea* latex	0.0404	0.0018	< 0.001*	0.0358 to 0.0450
		5.25% Sodium hypochlorite	–0.0012	0.0018	0.79	–0.0058 to 0.0034
	10% *C. gigantea* latex	5.25% Sodium hypochlorite	–0.0416	0.0018	< 0.001*	–0.0462 to –0.0370
After 15 minutes	5% *C. gigantea* latex	10% *C. gigantea* latex	0.0290	0.0031	< 0.001*	0.0213 to 0.0367
		5.25% Sodium hypochlorite	–0.0106	0.0031	0.006*	–0.0183 to –0.0029
	10% *C. gigantea* latex	5.25% Sodium hypochlorite	–0.0396	0.0031	< 0.001*	–0.0473 to –0.0319

* *P* < 0.05 indicates statistical significance. HSD: honestly significant difference.

## Discussion

This study demonstrated no significant difference in baseline pulp tissue weights among the experimental groups. Following immersion, tissue dissolution increased significantly with time in all groups. The 10% *C. gigantea* latex group showed the greatest weight reduction at both 10 and 15 minutes, with significantly greater dissolution than both 5% latex and 5.25% NaOCl. No significant difference was observed between 5% latex and NaOCl at 10 minutes, while a smaller but significant difference appeared at 15 minutes. These findings indicate a concentration- and time- dependent tissue-dissolving effect of the latex under standardised static conditions.

The efficacy of *C. gigantea* latex could be attributed to its rich enzymatic and phytochemical composition. A study by Ishnava et al. documented the presence of cysteine proteases, glycosides and flavonoids in *C. gigantea* latex, which is attributed to its protein-degrading capabilities and antimicrobial properties [16]. Saher et al. recently isolated a soluble laticifer protein from *Calotropis* species which demonstrated high proteolytic activity and antimicrobial action, providing plausibility for tissue-dissolving properties. Its biocompatibility for intra-oral usage has been validated by the study done by Sharma and Singh et al. in their anticariogenic mouth rinse study [12].

In endodontic practice, NaOCl is the gold standard irrigant for organic tissue dissolution due to its chlorination and saponification of proteins and amino acids [17]. A Brazilian study by Niewierowski et al. demonstrated that NaOCl solution (with and without ultrasonic agitation) was capable of complete pulp tissue dissolution and that ultrasonic activation significantly accelerated the process [18]. In the present study, under static conditions, 10% *C. gigantea* latex demonstrated dissolution comparable or greater than NaOCl, suggesting enzymatic proteolysis as a potential alternative mechanism rather than chemical oxidation alone. The efficacy of NaOCl is known to increase with agitation, ultrasonic activation, temperature elevation, and enhanced fluid dynamics. Both NaOCl and *C. gigantea* latex were evaluated under standardised static immersion conditions without activation. Therefore, the observed tissue dissolution reflects only their inherent chemical and enzymatic activity within a laboratory model and should be interpreted as preliminary *in vitro* evidence rather than clinical performance. No clinical superiority over NaOCl can be inferred.

Khademi et al. demonstrated the time-dependency of tissue dissolution of NaOCl, which showed that prolonged contact improved tissue removal, similar to the present study [17]. Irala et al. also demonstrated that higher NaOCl concentration resulted in faster dissolution than lower concentration, which aligns with the present study, where the 10% latex solution showed greater tissue-dissolving properties than the 5% solution [19]. These findings collectively indicate that both the concentration and exposure duration are critical determinants of tissue-dissolution efficacy, irrespective of the irrigant source.

Apart from tissue dissolution, a clinically effective irrigant must also eliminate persistent endodontic pathogens, particularly *Enterococcus faecalis*. Sahebi et al. reported that *Aloe vera* reduced *E. faecalis* counts but was significantly less effective than NaOCl, demonstrating clear intergroup antimicrobial differences [20]. Similarly, Karkare et al. found that garlic and *Aloe vera* extracts exhibited antimicrobial activity but remained inferior to NaOCl [21]. Studies evaluating *Propolis* and *Triphala* also showed meaningful antimicrobial action yet lower efficacy than NaOCl [22]. Conversely, *Moringa oleifera* extract has been shown to produce anti-bacterial activity comparable to NaOCl indicating that certain plant-derived agents can achieve clinically relevant anti-microbial performance [23]. Studies by Kaliamoorthy et al. and Bhavsar et al. demonstrated substantial bacterial reduction using herbal irrigants against *E. faecalis* biofilms, though NaOCl frequently produced greater reduction [13, 14].

Together, these studies indicate that comparisons between herbal and chemical irrigants typically produce measurable biological differences rather than subtle variations. *Calotropis gigantea latex may possess dual functional potential as both an organic tissue dissolution and as an antimicrobial agent*. The present study is subject to certain limitations. The static immersion model employed does not adequately replicate the dynamic clinical environment of root canal irrigation, including continuous fluid exchange, apical vapour lock phenomena, and intracanal pressure variations. Furthermore, adjunctive activation methods such as ultrasonic or sonic agitation were not incorporated, although these modalities are known to influence irrigant penetration and dissolution kinetics.

The evaluation was confined to pulp tissue dissolution, without assessment of additional clinically relevant parameters such as smear layer removal, dentin surface alterations, biofilm disruption, or effects on the organic–inorganic interface. Cytotoxicity and changes in dentin microhardness were also not experimentally determined; therefore, conclusions regarding biocompatibility and structural impact remain inferential.

The *C. gigantea* latex was processed into an ethanolic extract rather than being used in its raw milky form. Due to the low viscosity and favourable diffusion properties of ethanol, the extract may demonstrate improved flow characteristics and enhanced interaction with pulp tissue, potentially facilitating better penetration into organic remnants. However, neither the viscosity of the extract nor its penetration into dentinal tubules was quantitatively evaluated. Furthermore, the detoxification process employed in this study was qualitative, and the enzymatic activity of the processed latex was not quantitatively assessed. This may influence the reproducibility of the findings. Future studies should incorporate analytical techniques such as gas chromatography or spectroscopic methods for more accurate characterisation.

Future research should include dynamic irrigation models and activation protocols (ultrasonic, sonic activation) to simulate clinical conditions. Quantitative cytotoxic analysis, dentin microhardness assessment and smear layer removal studies are necessary before clinical translation. Standardisation of latex preparation through biochemical characterisation and enzyme activity measurement is required to ensure consistency. Further *in vitro* cytotoxicity testing and *in vivo* biocompatibility studies are necessary before considering any potential clinical application of this extract as an endodontic irrigant. And also, animal studies and randomised clinical trials are required to validate safety and therapeutic effectiveness as an endodontic irrigant.

## Conclusion

Within the limitations of this study, we concluded that the 10% solution of *C. gigantea* latex demonstrated a higher tissue-dissolving ability than 5.25% NaOCl under static *in vitro* conditions. However, these findings should not be directly extrapolated to clinical practice. Further investigations evaluating activation protocols, cytotoxicity, dentin interaction, and antimicrobial efficacy in simulated and *in vivo* environments are necessary before considering its use as an endodontic irrigant.

## Data Availability

The datasets generated and/or analysed during the current study are available from the corresponding author on reasonable request.
